# The Genotoxicity of Caecal Water in Gilts Exposed to Low Doses of Zearalenone

**DOI:** 10.3390/toxins10090350

**Published:** 2018-09-01

**Authors:** Katarzyna Cieplińska, Magdalena Gajęcka, Adriana Nowak, Michał Dąbrowski, Łukasz Zielonka, Maciej T. Gajęcki

**Affiliations:** 1 Microbiology Laboratory, Non-Public Health Care Centre, ul. Limanowskiego 31A, 10-342 Olsztyn, Poland; kasiacieplinska@gmail.com; 2 Department of Veterinary Prevention and Feed Hygiene, Faculty of Veterinary Medicine, University of Warmia and Mazury in Olsztyn, Oczapowskiego 13, 10-718 Olsztyn, Poland; michal.dabrowski@uwm.edu.pl (M.D.); lukaszz@uwm.edu.pl (Ł.Z.); gajecki@uwm.edu.pl (M.T.G.); 3 Institute of Fermentation Technology and Microbiology, Lodz University of Technology, 90-924 Lodz, Poland; adriana.nowak@p.lodz.pl

**Keywords:** zearalenone, doses, caecal water, genotoxicity, pre-pubertal gilts

## Abstract

Zearalenone is a toxic low-molecular-weight molecule that is naturally produced by moulds on crops as a secondary metabolite. The aim of this study was to determine the genotoxicity of caecal water collected successively from the caecal contents of gilts exposed to low doses (LOAEL, NOAEL, and MABEL) of zearalenone. The experiment was performed on 60 clinically healthy gilts with average BW of 14.5 ± 2 kg, divided into three experimental groups and a control group. Group ZEN5 were orally administered ZEN at 5 μg/kg BW, group ZEN10—10 μg ZEN/kg BW and group ZEN15—15 µg ZEN/kg BW. Five gilts from every group were euthanized on analytical dates 1, 2, and 3. Caecal water samples for in vitro analysis were collected from the ileocaecal region. The genotoxicity of caecal water was noted, particularly after date 1 in groups ZEN10 and ZEN15 with a decreasing trend. Electrophoresis revealed the presence of numerous comets without tails in groups C and ZEN5 and fewer comets with clearly expressed tails in groups ZEN10 and ZEN15. The distribution of LLC-PK1 cells ranged from 15% to 20% in groups C and ZEN5, and from 30% to 60% in groups ZEN10 and ZEN15. The analysis of caecal water genotoxicity during exposure to very low doses of ZEN revealed the presence of a counter response and a compensatory effect in gilts.

## 1. Introduction

The symptoms and health (toxicological) consequences of exposure to high doses of most mycotoxins, in particular, zearalenone (ZEN), are generally well known [1,2]. Research conducted in the past decade demonstrated that exposure to low doses of the parent compound without modified mycotoxins can also lead to health problems [3]. Similar findings had been reported previously by members of the EFSA Panel on Contaminants in the Food Chain [4,5]. The above is confirmed by the hormesis paradigm [6,7]. Doses below the lowest observed adverse effect level (LOAEL) values [8,9] that produce subclinical symptoms of disease are referred to as no observed adverse effect levels (NOAEL) doses [10]. The lowest measurable dose that enters positive interactions with the host’s body in various stages of life is known as the minimal anticipated biological effect level (MABEL) dose [11]. The dose-response paradigm has been undermined by the low dose hypothesis. The above applies particularly to hormonally active compounds [12], including mycosteroids such as ZEN. The dose-response relationship is ambiguous, and it does not support direct and monotone extrapolation or meta-analysis of the risks (clinical symptoms or laboratory results) associated with the shift from a high to a low dose [13]. The concept of a minimal dose (MABEL) that induces a counter-intuitive response is garnering increasing interest in biomedical sciences. The mechanisms associated with the MABEL dose must be researched to quantify the relevant risks and final outcomes [14] in the decision-making process.

Most *Fusarium* mycotoxins are absorbed primarily in the proximal segment of the small intestine [15]. Intestinal fragments are characterized by high physiological variability. The duodenum and the jejunum have the lowest content of mucus glycoproteins, which maximizes the availability of digesta for intestinal walls [16] and, consequently, the body. Most carbohydrates are also absorbed in the proximal segment of the small intestine [17], which promotes absorption, accumulation, and, probably, biotransformation of mycotoxins in enterocytes [18,19].

The highest percentage content of ZEN was observed in the small intestine in early stages of exposure. On successive days of exposure, ZEN was also accumulated in the duodenum and the descending colon. During exposure to the parent compound (ZEN) only, biotransformation processes were not observed or were significantly inhibited in the porcine gastrointestinal tract [20]. The accumulation of ZEN in intestinal tissues began already in the first week of exposure.

According to research into the changes that accompany exposure to small ZEN doses [21], the mycotoxin can produce side effects that are difficult to predict. This uncertainty is associated with both the dose and the duration of exposure. The exposure to small doses often produces surprising effects: (i) The body fails to recognize the presence of undesirable substances, such as mycotoxins [22], and the underlying principle is similar to the T-regs theory [23], postulating that these cells do not respond to small amounts of infectious factors; (ii) Mycotoxin absorption increases during prolonged *per os* exposure to ZEN [20]; (iii) The compensatory effect [24] inhibits the analysed factors, and homeostasis is restored [13] despite ongoing exposure.

Minimal doses of undesirable substances such as ZEN can be used for preventive or even therapeutic purposes. The genotoxicity of various (perhaps threshold) doses of the administered compound should be studied to determine their mutagenic effects. Fleck et al. [25] and Pfeiffer et al. [26] compared the genotoxicity of estradiol (E_2_), estrone (E_1_), ZEN, and α-zearalenol in a cell-free system and found that these compounds had similar DNA-damaging potential to endogenous steroids, and that they enhanced the carcinogenic effects of endogenous estrogens. These results are difficult to apply to the results of in vivo studies, which investigate the correlations between the dose and DNA-damaging potential.

De Ruyck et al. [27] observed that various substances, including carcinogenic compounds, are accumulated in the bodily tissues of animals exposed to ZEN. The epithelium of the digestive tract is exposed first to the ingested low doses of ZEN [28,29,30,31]. The intestinal mucosa prevents antigens, including undesirable substances such as ZEN, commensal bacteria, and pathogens from penetrating deeper tissues [32]. In a study by Nowak et al. [33], ZEN administered *per os* at 40 µg/kg BW increased the genotoxicity of caecal water (CW), mainly in the sixth week of the experiment, in the proximal and distal segments of the large intestine. Genotoxicity also increased in the proximal part of the colon. The authors suggested that the slow transit of intestinal contents after exposure to ZEN [34] increased the risk of adverse changes, including carcinogenic changes, in tissues containing estrogen receptors [35].

Genotoxins such as ZEN can damage DNA [36]. In somatic cells, DNA damage can lead to somatic mutations and, consequently, malignant transformations. There is a wide variety of in vitro and in vivo genotoxicity tests supporting the detection of many terminal points of DNA damage or its biological consequences for eukaryotic cells, including in mammals [37]. These tests should be used to evaluate the safety of feedstuffs and detect the presence of undesirable substances, including mycotoxins, in animal feed.

The comet assay is one of the most popular techniques for detecting DNA damage. The test is highly sensitive, and it can be performed on a small number of cells. Several modifications of the comet assay have been proposed to detect various types of DNA damage. The alkaline comet assay detects a combination of DNA changes, including single- and double-strand breaks, and alkali-labile sites [37]. Several indicators are used in the comet test, but the percentage of DNA damage in the comet tail (%DNAT) is regarded as the most reliable indicator, because it covers the broadest range of DNA damage and is linearly correlated with the frequency of DNA breaks [37]. For this reason, %DNAT was the indicator of choice in this study.

The above arguments indicate that prolonged oral administration of a natural parent compound such as ZEN increases carry-over values in successive segments of the gastrointestinal tract and increases the concentration of ZEN in digesta [20]. The degree of exposure can be evaluated by analysing the contents of the distal fragment of the digestive tract. The results of such analyses constitute valuable inputs for dietary intervention studies [33]. Biological markers are used to detect the DNA-damaging potential and/or mutagenic effects in digesta sampled from different intestinal segments. The aim of this study was to evaluate the genotoxicity of CW collected successively from the caecal contents of gilts exposed to low (LOAEL, NOAEL, and MABEL) doses of ZEN.

## 2. Results

### 2.1. Experimental Feed

The analysed feed did not contain mycotoxins, or its mycotoxin content was below the sensitivity of the method (VBS). The concentrations of modified and masked mycotoxins were not analysed.

### 2.2. Clinical Observations

Clinical signs of ZEN mycotoxicosis were not observed throughout the experiment. However, changes in specific tissues or cells were frequently observed in analyses of selected biochemical parameters in samples collected from the same animals and in those animals’ growth performance. The results of these analyses were published in a different paper [38].

### 2.3. General Information

The percentage of DNA damage in the comet tail (%DNAT) of non-exposed cells (negative control, LLC-PK1 cells in DMEM/Ham’s F12 medium) was determined at 4.1% ± 0.7%. In cells exposed to 25 µM hydrogen peroxide (positive control), %DNAT reached 86.3% ± 2.3%.

### 2.4. Single Cell Gel Electrophoreses (Comet Assay)

**Figure 1:**
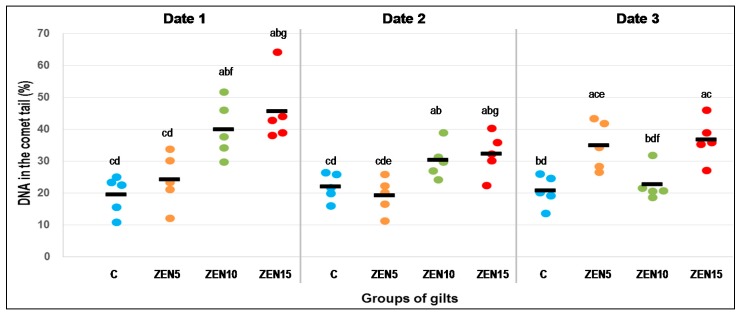
DNA damage expressed by the mean number of DNA breaks in the LLC-PK1 porcine kidney epithelial cell line of control gilts and gilts administered ZEN doses of 5, 10, and 15 μg ZEN/kg BW. Samples were collected on analytical dates 1, 2, and 3. A total of 100 cells was scored for each individual. Mean results differ significantly from: ^a^ control group, ^b^ ZEN5 group, ^c^ ZEN10 group, and ^d^ ZEN15 group values in a given week and ^e, f, g^ between the same concentrations of ZEN on different dates; ANOVA (*p* ≤ 0.05).

The genotoxicity of CW (Figure 1) obtained from gilts exposed to different ZEN doses was determined on three analytical dates. On every date, CW genotoxicity was lowest in group C (control group) with mean values of 19.5% to 22.0% and standard error of the mean (S.E.M.) of 2.7% and 1.9%, respectively.

No significant differences (Figure 1) were observed on analytical date 1 between groups C and ZEN5 (5 μg ZEN/kg BW), whereas the differences between groups ZEN10 (10 μg ZEN/kg BW) and ZEN15 (15 μg ZEN/kg BW) were significant. Similar differences were noted on date 2. On date 3, significant differences were observed between group C and groups ZEN5 and ZEN15 and between groups C and ZEN10 and groups ZEN5 and ZEN15.

Significant differences (Figure 1) between analytical dates were noted between groups ZEN10 and ZEN15 on date 1 vs. group ZEN10 on date 3 and group ZEN15 on date 2, and between group ZEN5 values on analytical dates 2 and 3.

**Figure 2:**
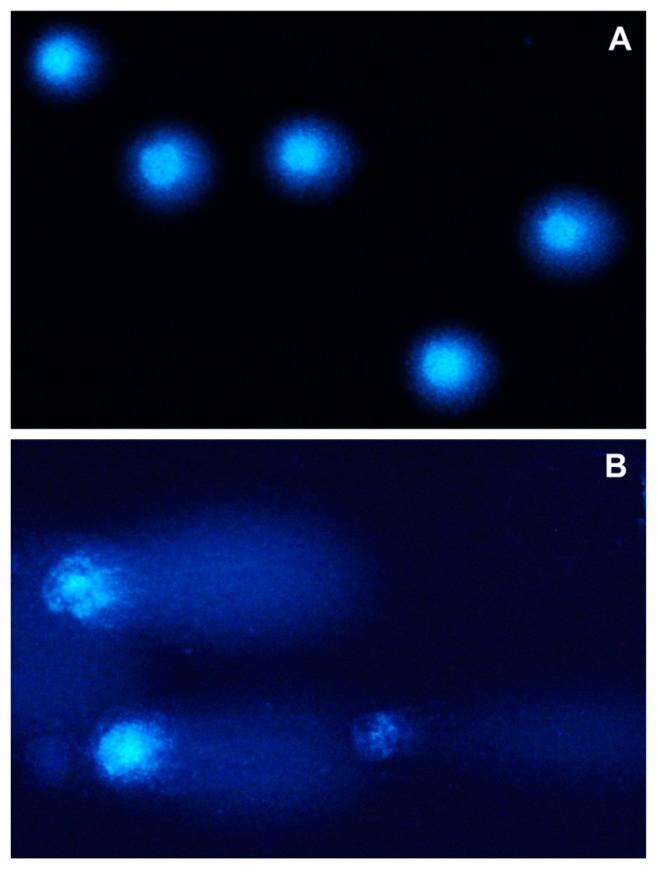
Typical images of DAPI-stained (4′,6-diamidino-2-phenylindole) comets: (**A**) group C; and (**B**) group ZEN15 on analytical date 1.

Significant differences (*p* ≤ 0.05) are indicative of genotoxicity (Figure 2—clear comet tail). On analytical date 1, an increase was observed in groups ZEN10 and ZEN15, in which mean values reached 40.0% ± 7.5% and 45.6% ± 7.4%, respectively, relative to groups C and ZEN5 (Figure 1), in which mean values were determined 19.8% ± 5.44% and 24.1% ± 6.26%, respectively.

On analytical date 2, %DNAT values were significantly less scattered across groups, but they were significantly more scattered in groups ZEN10 and ZEN15 (30.2% ± 3.9% and 32.2% ± 4.7%, respectively) relative to group C (21.8% ± 3.15%) and even more scattered relative in group ZEN5 (19.2% ± 4.25%) (*p* ≤ 0.05). These data indicate that genotoxic processes took place in groups ZEN10 and ZEN15, but not in groups C or ZEN5.

On analytical date 3, %DNAT was determined at 20.7% ± 3.67% in group C. In the LLC-PK1 cell line, %DNAT values in group ZEN5 increased in the range of 26.5% ± 3.3% to 43.3% ± 3.3%, with a mean value of 34.9% ± 6.2%. In group ZEN10, mean genotoxicity was determined at 22.6% ± 3.7% %DNAT, and it was lower than on dates 1 and 2 (40.0% ± 7.5% and 30.2% ± 3.9%, respectively) (*p* ≤ 0.05). In group ZEN15, %DNAT ranged from 27.0% ± 9.6% to 45.9% ± 9.34% (Figure 1), with a mean value 36.6% ± 4.7%.

The values of correlation coefficients (Pearson’s *r*) (Table 1) were calculated based on the relationship between the percentage of cells with damaged DNA and the percentage of DNA damage in a cell on different analytical dates and in different groups.

The results presented in Table 1 should be analysed in view of the strength of the observed correlations [39]. The correlation is positive when *r* > 0. The strength of correlations is evaluated on the following scale: *r* < 0.2—absence of a linear correlation; *r* = 0.2–0.4—weak correlation; *r* = 0.4–0.7—moderate correlation; *r* = 0.7–0.9—relatively strong correlation; and *r* > 0.9—very strong correlation. A weak correlation and a moderate correlation were noted in group C on two dates (very low %DNAT, which is natural). In group ZEN5, a weak correlation was observed initially, followed by an absence of linear correlations on two successive dates (very low %DNAT, which could suggest that the analysed ZEN dose has protective effects). A moderate correlation was noted in group ZEN10 and in group ZEN15, but an absence of a linear correlation was noted on date 2 in group ZEN15. Very low values of *r* in groups C and ZEN5 point to the presence of certain trends with some outliers. Genotoxicity was confirmed when the values of *r* were closer to 1. The above was noted in group ZEN15, but the observed trend was highly variable.

The electrophoresis of exposed cells revealed an absence of comet tails (even short tails) and nearly symmetrical comets in groups C and ZEN5 (Figure 2A). Groups ZEN10 and ZEN15 were characterized by comets with relatively long and long tails with damaged DNA, respectively (Figure 2B). These comets had very long tails and small heads. Their density (number of comets per slide) was considerably lower than in group C.

### 2.5. Histograms of Endogenous DNA Damage

**Figure 3:**
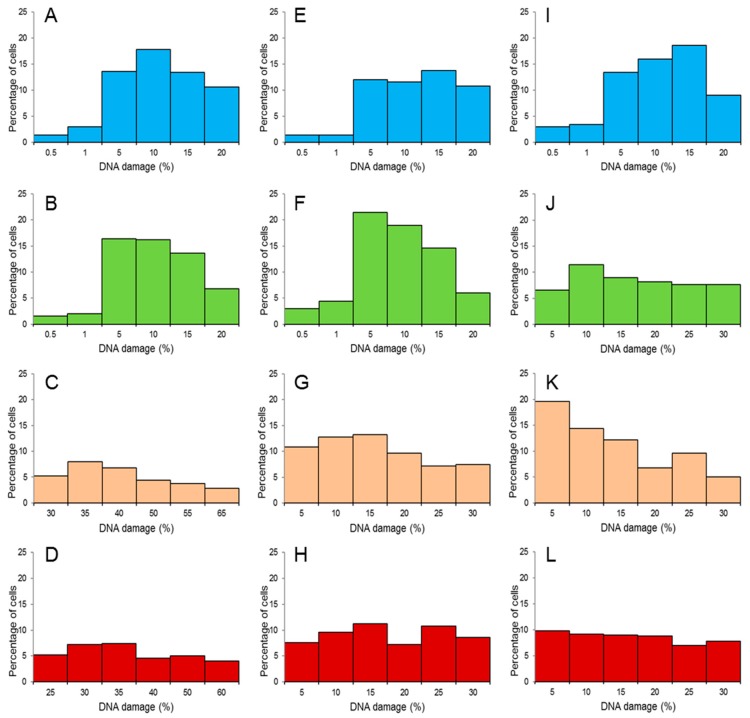
Histograms illustrating the distribution of endogenous DNA damage, measured as the mean %DNAT of porcine kidney epithelial cells (LLC-PK1 cell line) in: group C (**A**,**E**,**I**), CW of gilts fed with group ZEN5 (**B**,**F**,**J**), group ZEN10 (**C**,**G**,**K**) and group ZN15 (**D**,**H**,**L**). Analytical dates—date 1 (**A**–**D**), date 2 (**E**–**H**) and date 3 (**I**–**L**). A total of 100 cells were scored for each individual.

The distribution of LLC-PK1 cells based on the percentage of damaged DNA in the comet tail is presented in Figure 3. In group C, maximum %DNAT values were distributed in columns 5% or 10% on date 1 (Figure 3A), in columns 5% or 15% on date 2 (Figure 3E), and in columns 10% or 15% on date 3 (Figure 3I). The maximum percentage of cells with damaged DNA was relatively low (below 20%).

The distribution of cells with damaged DNA was similar in group ZEN5, in columns 5–15% (Figure 3B,F,J). On the first two analytical dates, %DNAT values were distributed in columns 5% or 10% (Figure 3B,F), and on date 3–in columns 10% or 15% (Figure 3J). On date 2, the maximum number of cells with damaged DNA exceeded 20% (Figure 3F), but it was noted in column 5%.

In groups ZEN10 and ZEN15, the maximum percentage of cells was very low (<10) on analytical date 1 (Figure 3C,D), but maximum %DNAT was distributed in columns 35% or 40% in group ZEN10 and in columns 30% or 35% in group ZEN15 (Figure 3C,D, respectively). In group ZEN10, the maximum percentage of cells with damaged DNA increased to 14% on date 2 (Figure 3G) and to nearly 20% on date 3 (Figure 3K). The maximum %DNAT values were distributed in columns 10% or 15% on date 2 (Figure 3G) and in columns 5% or 10% on date 3 (Figure 3K). In group ZEN15, the maximum percentage of cells with damaged DNA was estimated at 10% on analytical dates 2 and 3 (Figure 3H,L, respectively). The maximum %DNAT values were distributed in columns 15% or 25% on date 2 (Figure 3H) and in columns 5% or 10% on date 3 (Figure 3L).

## 3. Discussion

Zearalenone ingested with feed can lead to endocrinological disorders in humans and animals. Zearalenone is a macrocyclic β-resorcylic acid lactone with distinctive estrogen activity. Previous studies into the metabolism of ZEN revealed the presence of reducing metabolites, in particular α-zearalenol and its stereoisomer β-zearalenol. During the synthesis of these catechol metabolites, ZEN activity becomes similar to that of endogenous estrogens E_2_ and E_1_. These promiscuous steroids [40] and steroid-like substances are associated with the risk of bowel cancer [41], breast cancer [35], uterine cancer [9], and malignant changes in other estrogen-sensitive organs [21]. There is evidence to indicate that E_2_/E_1_ catechol metabolites are weak genotoxins that contribute to malignant transformations by producing quinones that generate DNA adducts [42]. Catechol production is the main metabolic pathway of ZEN and its metabolites in many mammals [43], including humans [44]. The above could suggest that even small doses of ZEN contribute to CW genotoxicity [25].

In this experiment, the tested ZEN doses confirmed that dose plays an important role during exposure to undesirable compounds. The above is validated by the results of the statistical analysis, which revealed a significant increase (*p* < 0.05) in CW genotoxicity on different analytical dates. The only exception was date 1 (exposure day 7) when maximum %DNAT values reached 39.8% ± 4.0% in group ZEN10 and 45.6% ± 4.8% in group ZEN15. The above results were among the highest %DNAT values noted in the experiment. The distribution of LLC-PK1 cells in groups C and ZEN5 based on their %DNAT values in comet tails ranged from 15% to 20%, which is a very low result when negative control values are subtracted (4.1%). These findings could point to the absence of genotoxicity in group ZEN5.

Similar observations were made in an analysis of the correlations between the percentage of cells with damaged DNA to the percentage of DNA damage in a cell (Pearson’s *r*) (Table 1). On analytical date 1, *r* values were equally low in group ZEN5 and in group C, which could validate the hypothesis that small amounts of undesirable substances are not detected by the body (similarly to the T-regs theory, which postulates that T-regs do not respond to small amounts of infectious factors) [23]. Zearalenone was recognised by the body only on analytical date 3, as in group C. The proposed MABEL dose probably approximated threshold values [25,26]. It should also be noted that pre-pubertal gilts are characterised by physiological hypoestrogenism [21] when the demand for estrogens exceeds supply. As an exogenous estrogen, ZEN (mycoestrogen) can be utilized by the body to exert protective effects. Interestingly, *r* values in group ZEN5 continued to decrease on successive analytical dates (Table 1), which could indicate that the MABEL dose (5 μg ZEN/kg BW) exerted a protective effect on gilts throughout the experiment.

In contrast, the values of *r* observed in groups ZEN10 (10 μg/kg BW—NOAEL) and ZEN15 (15 μg/kg BW—LOAEL) on date 1 point to hyperestrogenism, namely, the presence of “free” ZEN in the body. Hyperestrogenism increases %DNAT values, namely, the percentage of comets with much longer tails and small heads (Figure 2B) whose density was significantly lower than in group C (Figure 2A). The above suggests that CW genotoxicity, preceded by oxidative damage and mitotic spindle dysfunction, is induced already by the NOAEL dose (10 μg ZEN/kg BW) [42]. The above is generally accompanied by a host of genotoxic effects in CW, including intensified Ca^2+^ transfer to cells [45] and changes in the activity of selected hydroxysteroid dehydrogenase enzymes [46], which can lead to steroidogenesis disorders [19].

The ZEN doses administered to groups ZEN10 and ZEN15 induced CW genotoxicity, but the observed correlations were moderate (*r* = 0.4 to 0.7, Table 1) with a decreasing trend (Figure 3). According to Błasiak et al. [47], the results of the comet assay should be analysed based on the distribution of cells based on damage to their DNA (histograms) to determine the susceptibility of individual cells to DNA damage. The histograms illustrating the distribution of damage to endogenous DNA (Figure 3G,H,K,L) in the experimental cells differ significantly between groups. On analytical date 1, the histograms for groups ZEN10 and ZEN15 (Figure 3C,D) were very strongly shifted toward higher values (from 20% to 65%). On date 2, the extent of DNA damage decreased 1.3- to 1.4-fold relative to date 1. On the last two analytical dates, the histograms for groups ZEN10 and ZEN15 were significantly flattened and shifted toward higher values in comparison with the histograms for groups C and ZEN5 (Figure 3H,L). This could imply that under exposure to higher ZEN doses, genotoxic processes in CW are initially more expressed and can provoke clinical symptoms. On successive days of exposure, genotoxic processes in CW were still more pronounced than in groups C and ZEN5. The above could be attributed to the fact that in gilts exposed to ZEN, the carry-over of ZEN to tissues other than the intestines [20], the initiation of biotransformation processes in the liver, the detoxifying activity of gut microbiota [48], and the conversion of modified mycotoxins to a free form in the colon [3] proceeded at a faster rate than the destruction of DNA in the caecal epithelium.

The results of this study suggest that ZEN has a damaging effect on cells cultured in vitro and, most importantly, on cells on the organism of gilts. The values corresponding to the percentage of DNA breaks in cells were highly scattered. On successive days of exposure to ZEN, damage histograms were flattened and shifted toward lower values in groups C and ZEN5 and were shifted toward higher values in groups ZEN10 and ZEN15, which indicates that the counter response in the porcine model is influenced by the amount of ZEN ingested with feed.

The present results support the formulation of two hypotheses. The first hypothesis postulates the presence of a compensatory effect based on the values of *r* in groups ZEN10 and ZEN15, which were higher on analytical date 1 than on successive days of exposure. The second hypothesis states that the proposed MABEL dose is not genotoxic, but further research is needed to confirm this observation.

## 4. Conclusions

The results of this study, which investigated CW genotoxicity, indicate that very small doses of ZEN (LOAEL, NOAEL, and MABEL) induce a counter response and a compensatory effect in the porcine model. The extent of DNA damage was proportional to the administered mycotoxin dose. Our findings suggest that the MABEL dose could be used for preventive purposes in pre-pubertal gilts.

## 5. Materials and Methods

### 5.1. In Vivo Study

#### 5.1.1. General Information

All experimental procedures involving animals were carried out in compliance with Polish regulations setting forth the terms and conditions of animal experimentation (Opinions No. 12/2016 and 45/2016/DLZ of the Local Ethics Committee for Animal Experimentation of 27 April 2016 and 30 November 2016).

#### 5.1.2. Experimental Animals and Feed

The in vivo experiment was performed at the Department of Veterinary Prevention and Feed Hygiene of the Faculty of Veterinary Medicine at the University of Warmia and Mazury in Olsztyn on 60 clinically healthy pre-pubertal gilts with initial BW of 14.5 ± 2 kg. The animals were housed in pens with free access to water. All groups of gilts received the same feed throughout the experiment. They were randomly assigned to three experimental groups (group ZEN5, group ZEN10, and group ZEN15; *n* = 15) and a control group (group C; *n* = 15) [49,50]. Group ZEN5 gilts were orally administered ZEN (Z2125-26MG, Sigma-Aldrich, St. Louis, MO, USA) at 5 μg ZEN/kg BW, group ZEN10 pigs–10 μg ZEN/kg BW, and group ZEN15 pigs–15 μg ZEN/kg BW. Analytical samples of ZEN were dissolved in 96 µL of 96% ethanol (SWW 2442-90, Polskie Odczynniki SA, Poland) in weight-appropriate doses. Feed containing different doses of ZEN in an alcohol solution was placed in gel capsules. The capsules were stored at room temperature before administration to evaporate the alcohol. In the experimental groups, ZEN was administered daily in gel capsules before morning feeding. The animals were weighed at weekly intervals, and the results were used to adjust individual mycotoxin doses. Feed was the carrier, and group C pigs were administered the same gel capsules but without mycotoxins.

The feed administered to all experimental animals was supplied by the same producer. Friable feed was provided *ad libitum* twice daily, at 8:00 a.m. and 5:00 p.m., throughout the experiment. The composition of the complete diet, as declared by the manufacturer, is presented in Table 2.

The proximate chemical composition of diets fed to pigs in groups C, ZEN5, ZEN10, and ZEN15 was determined using the NIRS™ DS2500 F feed analyser (FOSS, Hillerød, Denmark), a monochromator-based NIR reflectance and transflectance analyser with a scanning range of 850–2500 nm.

#### 5.1.3. Toxicological Analysis

Feed was analysed for the presence of ZEN and DON by high-performance liquid chromatography with UV–vis detection (HPLC-UV). The obtained values did not exceed the limits of quantitation (LOQ) of 2 ng/g for ZEN and 5 ng/g for DON based on the validation of chromatographic methods for the determination of ZEN and DON levels in feed materials and feeds, which was performed at the Department [51].

#### 5.1.4. Sampling for In Vitro Tests

Five gilts from every group were euthanized on analytical date 1 (exposure day 7), date 2 (exposure day 21), and date 3 (exposure day 42) by intravenous administration of pentobarbital sodium (Fatro, Ozzano Emilia BO, Italy) and bleeding. Sections of intestinal tissues were collected immediately after cardiac arrest and were prepared for analyses. Caecal water samples for in vitro analysis were collected from a 10-cm-long intestinal fragment resected from the ileocecal region and the colon. Intestinal segments were tied at both ends before resection to avoid tissue damage. The resected fragments were transported to the laboratory, and CW samples were analysed.

### 5.2. In Vitro Study

The in vitro experiment was performed at the Institute of Fermentation Technology and Microbiology, Lodz University of Technology in Lodz.

#### 5.2.1. Caecal Water Preparation

Caecal water samples were collected in plastic containers. Freshly obtained caecal contents (20%) were mixed with sterile phosphate-buffered saline (PBS, pH 7.2) (80%), homogenised for 2 min, and centrifuged (10,700× *g*, 40 min, 4 °C). The supernatant fractions were filtered (0.45 µm pore size, Merck–Millipore, Darmstadt, Germany) and stored at −20 °C until analysis.

#### 5.2.2. Cell Culture and Treatment

The LLC-PK1 porcine kidney epithelial cell line (Cell Lines Service, Eppelheim, Germany) from 38 passage was used in the research. This cell line is often used to test the genotoxicity and cytotoxicity of mycotoxins [36,52].

The cells were cultured in T75 flasks (Roux type) (Becton, Dickinson and Co., Franklin Lakes, NJ, USA) as a monolayer in Dulbecco’s Modified Eagle’s Medium/Ham’s F12 (DMEM/Ham’s F12, 1:1; Cell Lines Service, Eppelheim, Germany) with 5% foetal bovine serum (FBS, Cell Lines Service, Eppelheim, Germany), supplemented with 2 mM l-glutamine (Cell Lines Service, Eppelheim, Germany) and HEPES (Cell Lines Service, Eppelheim, Germany). The cells were incubated in a CO_2_ incubator (Galaxy 48S, New Brunswick, UK) at 37 °C under 5% CO_2_ atmosphere for 7 days, until confluence. The cells were sub-cultivated, and the medium was changed every 2–3 days. LLC-PK1 cells were detached with TrypLE^TM^ Express (Gibco, Thermo Fisher Scientific, Waltham, MA, USA) for 20 min and gently shaken off the plastic flask. According to the manufacturer’s instructions, neutralisation with FBS is not required for reagents of the plant origin. After detaching, the cell suspension in PBS (Sigma-Aldrich, St. Louis, MO, USA) was transferred to a Falcon tube, centrifuged (182× *g*, 5 min), decanted, and resuspended in fresh medium. Cell counts and viability were determined by trypan blue exclusion assay (min. 90%), and the cells were ready to use.

#### 5.2.3. Comet Assay—Single Cell Gel Electrophoresis Assay (SCGE)

The final concentration of LLC-PK1 cells in each sample was adjusted to 10^5^ cells/mL. The cells were incubated with CW (20%, *v/v*) at 37 °C for 1 h. The cells were incubated in a medium without FBS to avoid interactions between FBS and the mycotoxin.

The comet assay was performed under alkaline conditions (pH > 13) according to Błasiak and Kowalik [53], as described previously [33]. After incubation, the cells were centrifuged (182× *g*, 15 min, 4 °C), decanted, suspended in 0.75% low melting point (LMP) agarose (Sigma-Aldrich, St. Louis, MO, USA), layered onto slides precoated with 0.5% normal melting (NMP) agarose (Sigma-Aldrich, St. Louis, MO, USA), and lysed at 4 °C for 1 h in a buffer consisting of: 2.5 M NaCl, 1% Triton X-100, 100 mM EDTA and 10 mM Tris, pH 10. Next, the slides were placed in an electrophoresis unit, and DNA was allowed to unwind for 20 min in an unwinding buffer containing 300 mM NaOH and 1 mM EDTA. Electrophoresis was conducted at 4 °C for 20 min at field strength of 0.73 V/cm (300 mA) in electrophoretic buffer containing 30 mM NaOH and 1 mM EDTA. Then, the slides were neutralised (0.4 mol/L Tris), stained with 1 µg/mL 4′,6-diamidino-2-phenylindole (DAPI, Sigma-Aldrich, St. Louis, MO, USA), and covered with cover slips. The slides were analysed at 200× magnification under a fluorescence microscope (Nikon, Tokyo, Japan) connected to a digital camera (Nikon Digital Sight DS-U3, Tokyo, Japan) and the Lucia-Comet v. 7.0 digital image analysis system (Laboratory Imaging, Prague, Czech Republic). One hundred images were randomly selected from each sample, and %DNAT was determined as a measure of DNA damage.

#### 5.2.4. Statistical Analysis

Comet assay data were analysed by two-way analysis of variance (ANOVA), and mode of interaction × time was used to compare the effects induced by the chemicals in the analysed mode of interaction. Differences between samples with normal distribution were evaluated. ANOVA was performed using OriginPro 6.1 software (OriginLab Corporation, Northampton, MA, USA). Dose–response relationships were determined by Pearson’s correlation. Differences were regarded as significant at *p* ≤ 0.05. The results were presented as means ± standard error of the mean (S.E.M.).

## Figures and Tables

**Table 1:** Correlation coefficients (Pearson’s *r*).

Analytical Date	Group C	Group ZEN5	Group ZEN10	Group ZEN15
1	0.29	0.29	0.52	0.61
2	0.45	0.16	0.41	0.08
3	0.26	0.07	0.44	0.46

Key: Ratio between the percentage of cells with damaged DNA and the percentage of DNA damage in a cell on different analytical dates and in different groups: Group C—placebo; Group ZEN5—5 μg ZEN/kg BW, administered once daily before morning feeding; Group ZEN10—10 μg ZEN/kg BW, administered once daily before morning feeding; Group ZEN15—15 μg ZEN/kg BW, administered once daily before morning feeding. Samples were collected immediately after euthanasia on three analytical dates (exposure days 7, 21, and 42).

**Table 2:** Declared composition of the complete diet.

Parameters	Composition Declared by the Manufacturer (%)
Soybean meal	16
Wheat	55
Barley	22
Wheat bran	4.0
Chalk	0.3
Zitrosan	0.2
Vitamin-mineral premix ^1^	2.5

^1^ Composition of the vitamin-mineral premix per kg: vitamin A–500.000 IU; iron–5000 mg; vitamin D3–100.000 IU; zinc–5000 mg; vitamin E (alpha-tocopherol)–2000 mg; manganese–3000 mg; vitamin K–150 mg; copper (CuSO_4_·5H_2_O)–500 mg; vitamin B_1_–100 mg; cobalt–20 mg; vitamin B_2_–300 mg; iodine–40 mg; vitamin B_6_–150 mg; selenium–15 mg; vitamin B_12_–1500 μg; L-lysine–9.4 g; niacin–1200 mg; DL-methionine+cystine–3.7 g; pantothenic acid–600 mg; L-threonine–2.3 g; folic acid–50 mg; tryptophan–1.1 g; biotin–7500 μg; phytase+choline–10 g; ToyoCerin probiotic+calcium–250 g; antioxidant + mineral phosphorus and released phosphorus–60 g; magnesium–5 g; sodium; calcium–51 g.
